# Using existing trial data to inform the development of core outcome sets and improve efficiencies in research

**DOI:** 10.1186/1745-6215-16-S2-O74

**Published:** 2015-11-16

**Authors:** Kim S Thomas, Lucy Bradshaw, Alan A Montgommery, Nick A Francis, Matthew J Ridd, Miriam Santer

**Affiliations:** 1 Centre of Evidence Based Dermatology, University of Nottingham, Nottingham, UK; 2 Nottingham Clinical Trials Unit, University of Nottingham, Nottingham, UK; 3 Cochrane Institute of Primary Care and Public Health, Cardiff, Wales, UK; 4 Centre for Academic Primary Care, School of Social and Community Medicine, Bristol, UK; 5 Department of Primary Care and Population Sciences, University of Southampton, Southampton, UK

## Introduction

One way of improving efficiency of research is to use core outcome sets, which allow results from different trials to be compared. Obtaining consensus over core outcome sets requires evidence on the validity, reliability and responsiveness of chosen measurement instruments. Re-using existing trial datasets to conduct validation studies of measurement instruments could be an efficient and timely means of filling known validation gaps.

## Methods

Using an exemplar from the field of eczema, evidence presented at meetings of the Harmonizing Outcome Measures for Eczema (HOME) initiative (http://www.homeforeczema.org) was reviewed, and included: qualitative surveys (content validity); systematic reviews of existing trials (to identify preferred outcome instruments); and systematic reviews of validation studies (to identify validation gaps). Known validation gaps were identified and mapped to five existing trial datasets, to establish which gaps could feasibly be filled using existing primary data (NIHR-funded eczema trials: (SWET - HTA:05/16/01, CLOTHES - HTA:11/65/01, CREAM - HTA:09/118/03, BATHE - HTA:11/153/01, COMET - RfPB: PB-PG-0712-28056).

## Results

The five included trials contain data on outcome instruments relating to all four core outcome domains for eczema. Results will be presented to show how existing trial datasets have been used to address validation gaps including construct validity (structural validity and hypothesis testing), reliability (internal consistency) and responsiveness to change. Trial data have also been used to help define the construct of long-term control; particularly the definition of eczema flares and optimum frequency of data collection.

## Conclusions

Re-using existing trial datasets to inform core outcome set development is feasible, efficient and timely.

